# Zoom in on neurodegeneration

**DOI:** 10.1186/1750-1326-1-1

**Published:** 2006-06-12

**Authors:** Guojun Bu, Huaxi Xu, Todd E Golde

**Affiliations:** 1 Departments of Pediatrics, and Cell Biology and Physiology, Washington University School of Medicine, St Louis, MO 63110, USA; 2 Center for Neuroscience and Aging, The Burnham Institute, La Jolla, California 92037, USA; 3 Department of Neuroscience, Mayo Clinic, Mayo Clinic College of Medicine, Jacksonville, Florida 32224, USA

## Abstract

Despite the existence of several scientific journals that publish research papers and reviews related to neurodegenerative diseases, a journal specifically devoted to the molecular and cellular aspects of disease mechanisms is lacking. *Molecular Neurodegeneration *is an open-access, peer-reviewed, online journal created to publish original research articles that address i) the mechanisms of neurodegeneration at the cellular, subcellular and molecular levels and ii) potential therapeutic interventions for neurodegenerative diseases. Through publication of reviews, editorial commentaries, and meeting reports, *Molecular Neurodegeneration *will also provide a forum to enhance the exchange of ideas and promote debate that is essential for scientific progress. *Molecular Neurodegeneration *will enable scientists to rapidly communicate their important research discoveries to their colleagues around the world.

## 

We are proud to introduce you to a new home for neurodegeneration research, *Molecular Neurodegeneration*. Through the open-access format we plan to bring you the most timely and exciting research focusing on the molecular and cellular mechanisms underlying neurodegenerative disease processes and on potential therapeutic interventions for these devastating diseases. *Molecular Neurodegeneration *will also provide a forum to discuss and review the current research and foster creative thinking in this exciting field.

The increased prevalence of certain age-associated neurodegenerative diseases is largely attributable to the increase in average lifespan among individuals who live in industrialized nations. From a patient perspective, diseases such as Alzheimer's, Parkinson's or amyotrophic lateral sclerosis are feared because of their slow and progressive nature and because there are no effective treatments or cures for these diseases. The economic and social burden of neurodegenerative diseases is huge and growing all too rapidly. We have made significant progress with respect to understanding certain aspects of neurodegenerative diseases. However, compared to our understanding of other human diseases such as cancer and cardiovascular disorders, our knowledge on the mechanisms of neurodegeneration is still in its infancy. Indeed, for most neurodegenerative diseases, we can only guess as to why neurons ultimately die.

Most existing scientific journals that publish research papers related to neurodegenerative diseases focus on the genetic epidemiology and pathological aspects of these diseases. *Molecular Neurodegeneration *will uniquely focus on the molecular and cellular aspects of the disease mechanisms, identification of potential therapeutic targets, and preclinical studies evaluating potential therapeutic interventions in model systems. Studies of the physiological and pathophysiological functions of cellular proteins contributing to neurodegeneration are strongly encouraged. We hope this focus will be particularly attractive to neurodegenerative disease researchers whose interests are at the molecular, subcellular and cellular levels as well as those who are trying to develop and evaluate novel "target-based" therapies for these diseases.

The majority of published manuscripts will be research articles and reviews. However, editorials, short reports, commentaries, meeting reports, and articles concerning methodology will also be considered. Each manuscript will be peer reviewed to ensure that the highest standards are maintained. Once accepted, papers will be immediately published online and will be listed in PubMed.

An important feature of *Molecular Neurodegeneration *is the open-access format. Traditional journals are often access-restricted and open only to those universities, research institutes, and individuals who can afford the high fees for electronic access. We hope that the open-access format will be attractive to scientists around the world because of its broad readership. From an editorial perspective we will strive to ensure that manuscripts are reviewed in both a fair and timely fashion.

The debut of *Molecular Neurodegeneration *would not have been possible without the strong support of many scientists who are at the forefront of research in neurodegeneration. *Molecular Neurodegeneration *has a strong editorial board with a wide range of expertise in different neurodegenerative diseases and different approaches to disease mechanisms. Members of *Molecular Neurodegeneration's *editorial board are committed to support of the journal by helping to identify important and interesting research manuscripts, serving as reviewers, and contributing their own work to the journal. Our goal is for *Molecular Neurodegeneration *to publish novel findings that can advance the field of neurodegeneration research. We hope for enthusiastic support from scientists around the world and welcome suggestions that will make *Molecular Neurodegeneration *an important resource in this critical field.

## Competing interests

The author(s) declare that they have no competing interests.

